# Changes in the distribution of mechanically dependent plants along a gradient of past hurricane impact

**DOI:** 10.1093/aobpla/plv096

**Published:** 2015-08-18

**Authors:** Sven P. Batke, Daniel L. Kelly

**Affiliations:** 1 Department of Botany, Trinity College, The University of Dublin, College Green, Dublin 2, Ireland; 2 Trinity Centre for Biodiversity Research, Trinity College, The University of Dublin, Dublin 2, Ireland; 3 Operation Wallacea, Hope House, Old Bolingbroke, Lincolnshire PE23 4EX, UK

**Keywords:** Cusuco National Park, disturbance, epiphyte, plant community, species diversity, species richness

## Abstract

The severity of the effects that large disturbance events such as hurricanes can have on the forest canopy and the associated mechanically dependent plant community (epiphytes, climbers, etc.) is dependent on the frequency and intensity of the disturbance events. Here we investigate the effects of different structural and environmental properties of the host trees and previously modelled past hurricanes on dependent plants in Cusuco National Park, Honduras. Tree-climbing methods were employed to sample different dependent life-forms in ten 150 × 150 m plots. We identified 7094 individuals of dependent plants from 214 different species. For holo- and hemi-epiphytes, we found that diversity was significantly negatively related to past hurricane impact. The abundance of dependent plants was greatly influenced by their position in tree canopy and hurricane disturbance regimes. The relationship between abundance and mean branch height shifts across a gradient of hurricane impact (from negative to positive), which might result from a combination of changes in abundance of individual species and composition of the dependent flora across sites. Mechanically dependent plants also responded to different structural and environmental conditions along individual branches. The variables that explained much of the community differences of life-forms and families among branches were branch surface area and bryophyte cover. The factors that explained most variation at a plot level were mean vapour pressure deficit and elevation. At the level of the individual tree, the most important factors were canopy openness and past hurricane impact. We believe that more emphasis needs to be placed on the effects that past disturbance events have on mechanically dependent plant communities, particularly in areas that are prone to catastrophic perturbations.

## Abstract (precis)

Past hurricane events have negatively affected the current diversity and composition of canopy dependent plant communities, researchers report. Using advanced climbing techniques, the team studied the distribution and composition of mechanically dependent plants (e.g. epiphytes, climbers etc.) on +45 m tall forest canopy trees in Honduras, and found that their diversity was significantly decreased on sites that had been more impacted by hurricanes. It was also found that the degree of their response varied at different scales (i.e. the plot, tree and branch level). These results are of great importance to understand the imminent and past impacts of hurricane storms on canopy communities in hurricane prone regions.

## Introduction

Disturbance is an important driver in landscape community ecology and can be summarized as ‘… any relatively discrete event in time that disrupts ecosystems, community or population structure and changes resources, substrate availability, or the physical environment’ (Pickett and White 1985). Any community has been and is shaped by past and present disturbance events (Smith *et al*. 2012). The spatial variation in the frequency and intensity of large disturbance events often results in a vegetation mosaic of different ages and successional classes (Tanner *et al*. 2014), with associated alterations in micro-environmental conditions (Turton and Siegenthaler 2004). Therefore, past effects of disturbance can produce gradients of vegetation and environmental conditions (Foster and Boose 1992) that often can be measured long after the passing of such events (Batke and Kelly 2014; Batke *et al*. 2014).

Hurricanes are one example of a disturbance event that can have devastating effects on a region (Basnet *et al*. 1992; Bellingham *et al*. 1992; Cahoon *et al*. 2003; Wagner *et al*. 2014). Many ecosystems have been altered structurally and biologically as a result of such high-energy weather events (Brokaw and Walker 1991; van der Maarel and Franklin 2012). For example, in forest systems, hurricane impact can result in tree blow-down, below- and above-ground gap formation, mineral and nutrient leaching and soil erosion (Tanner *et al*. 1991). Heartsill *et al*. (2010) demonstrated that above-ground biomass was reduced by 50 % following a Category 4 hurricane in Puerto Rico. Stem density and tree diversity were also significantly reduced. Although the immediate impact was severe, the forest recovery was rapid and it had almost returned to pre-hurricane structural conditions after 15 years. In most instances, research on disturbance from hurricanes has focussed on the immediate effects of damage but past effects of disturbances have been studied less (Tanner *et al*. 2014; Wagner *et al*. 2014). As many canopy-dwelling organisms are dependent on the forest canopy's long-term structural and environmental stability (Sillett and Antoine 2004), severe, repetitive damage from hurricanes is likely to affect the long-term composition and persistence of forest communities (Raventós *et al*. 2015).

Mechanically dependent plants (Kelly 1985)—epiphytes, climbers, etc.—are plants that depend on trees for physical support. The majority are detached from terrestrial resources and are thus highly dependent on the physical environment of the host tree (e.g. quality of the branch substratum) (Benzing 1987, 1990). Their tight coupling to the atmosphere makes them very vulnerable to any sudden changes in the structure and microclimate of the host tree (Callaway *et al*. 2002; Aguirre *et al*. 2010; Gehrig-Downie *et al*. 2011). For example, the dislodgement of branches from hurricanes has shown to increase light levels and temperature and decrease relative humidity (Boucher *et al*. 1990; Oberbauer *et al*. 1996; Turton and Siegenthaler 2004; Turton 2013). The extent to which mechanically dependent plants respond to such damage will depend on species-specific traits (ecophysiological and morphological) that enable them to tolerate or adapt to these changes (Rees *et al*. 2001), and to the underlying effects of the changed forest structure (Zimmerman *et al*. 2014) and host composition (Robertson and Platt 2001) following perturbation. For example, dependent plants that have high water-use efficiency, a robust photo-protective capacity and a rooting system that enables them to withstand strong winds are expected to become more prevalent in canopies that have been more frequently damaged by hurricanes (Goode and Allen 2008).

Little is known on the long-term response of mechanically dependent plant communities that have been affected by multiple hurricane events. It is also unclear how the diversity and distribution of these communities is altered along the vertical forest profile as a result of hurricane damage and the physical environment of the host tree. To answer these important questions, our study aims to investigate the responses of dependent plant community composition and diversity to different structural and environmental variables of the host tree, across a gradient of past hurricane impact. We used a hurricane model that was developed for Cusuco National Park (CNP), Honduras, which allowed us to identify sites that have been least/most impacted by hurricanes over a 15-year period (Batke *et al*. 2014). Our main research questions were: (i) Has the diversity and richness of mechanically dependent plants been altered between trees that were exposed to different levels of hurricane impact? (ii) Has their distribution and composition along the host tree been altered as a result of it? (iii) And what is the contribution of different environmental variables on their composition at a plot, tree and branch level?

## Methods

### Study site

**Figure 1.**
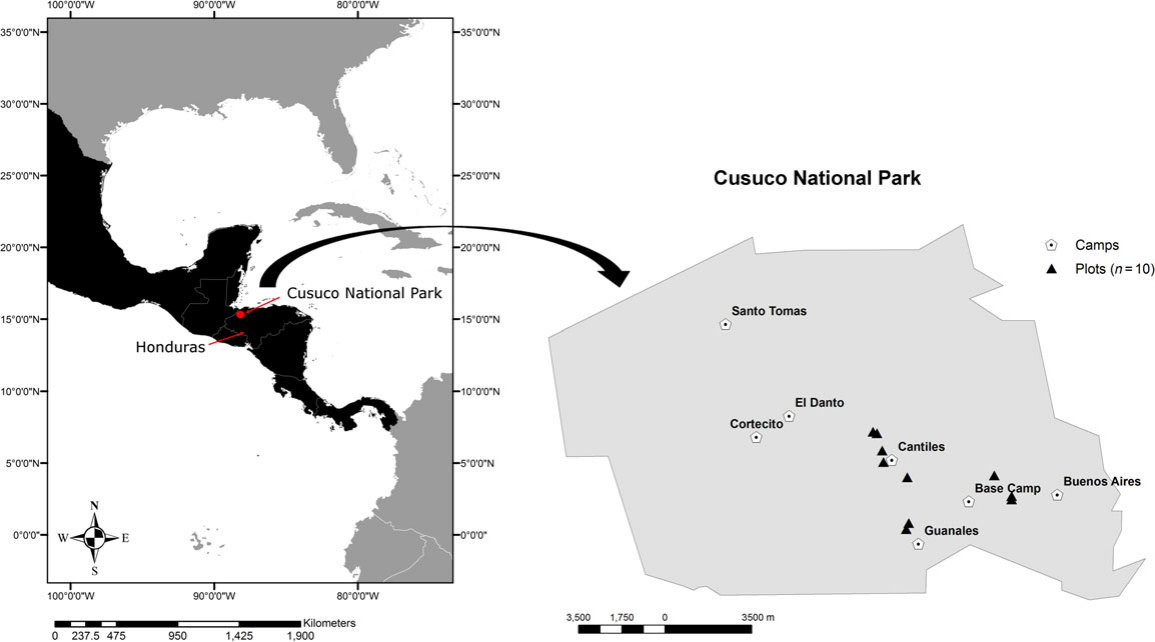
Location of our study sites in Cusuco National Park, Honduras.

Cusuco National Park (CNP) is located in the Departments of Santa Barbara and Cortés in north-west Honduras (15°32′31″N, 88°15′49″W; Fig. 1). See Batke *et al*. (2014) and Batke and Kelly (2014) for a more detailed description of the study site. Briefly, the mountain cloud forest is dominated by broadleaved and needle-leaved tree species from the families Pinaceae, Altingiaceae, Fagaceae, Melastomataceae, Lauraceae, Rubiaceae and Euphorbiaceae. Maximum elevation is 2242 m a.s.l., with annual precipitation of ∼2500 mm (Baker 1994).

### Plot and tree selection

Data for this study were collected within ten 150 × 150 m (2.25 ha) plots [see Batke *et al*. (2014) for more details]. The six largest trees (three *Pinus* spp. and three broadleaved angiosperms) within each plot were selected and subsequently surveyed for all vascular mechanically dependent taxa. Atypical and/or noxious tree types were avoided, notably palms, trees with flaky bark trees with irritant properties (*Toxicodendron*), ant-trees (*Cecropia*) and stranglers (*Ficus* spp.).

### Data collection

At a plot level, aspect, inclination and exposure within each plot were recorded [see Batke *et al*. (2014) for more details]. For trees that could not be identified in the field, samples were collected for later identification. Diameter at breast height at a standard height of 1.3 m or, where appropriate, above the top of the buttresses was recorded and tree height measured by climbing followed by lowering a tape measure. The horizontal extent of the canopy was estimated at ground level by measuring from the centre of the tree in the four cardinal directions (north, west, east and south). Tree surface area was calculated by measuring the length and circumference of each branch that was >10 cm circumference and treating the branch as a cylinder, as described by Batke (2012).

Canopy openness, i.e. the percentage area of the sky that is unobstructed by vegetation (Frazer *et al*. 1997), was visually estimated independently by three individual observers from the bottom of each tree. Openness was estimated from the north side of the tree, using a sighting angle of 75° to the top of the tree, with a distance of 5 m from the centre of the tree. ‘Closed’ was defined as where the cover of the tree canopy was >80 %. ‘Intermediate’ was defined as where the cover of the tree canopy was >20 and <80 %, and ‘open’ was defined as where the cover of the tree canopy was <20 %.

The within-tree data collection was undertaken using modern rope access methods. Mechanically dependent plants were divided into the following categories, following Zotz (2013) and Moffett (2000): holo-epiphytes, primary hemi-epiphytes (i.e. they germinate in the canopy and subsequently send roots down to the soil), nomadic vines (i.e. they germinate at or near ground level and climb upwards, subsequently losing their stem connection to the soil), climbers (i.e. vines; includes lianes), hemi-parasitic epiphytes (mistletoes) and accidental epiphytes. As little is still known about the ecology of many epiphytic and terrestrial plants within CNP, the terms obligate epiphyte (i.e. exclusively aerial) and facultative epiphyte (i.e. sometimes also terrestrial) were not used. Every branch and bole that hosted vascular-dependent plants was sampled. Abundance, life-form and fertility were recorded for each species. New species to the field collection were collected for subsequent identification. Branches that were difficult to access, usually when <35 cm circumference, were cut and lowered to the ground for assessment. For species with a vertical growth habit [i.e. compact epiphytes (Kelly 1985)], abundance was measured by counting the total number of individuals per branch (or bole). Juvenile stages were not included except for woody species. For species that spread laterally by rhizomes (and where individuals are consequently hard to define), abundance was quantified in terms of total area of each individual patch (i.e. length and width of the occupied area) and number of patches observed per branch. Specimens that were collected during the fieldwork were identified at the Cyril Hardy Nelson Sutherland Herbarium (TEFH: National Autonomous University of Honduras) and the herbaria at Trinity College Dublin (TCD), the Natural History Museum, London (BM) and the Royal Botanic Gardens, Kew (K). Specimens are lodged at TCD and TEFH.

At an individual tree level we recorded branch characteristics such as length and circumference of each branch, bark texture, aspect and inclination. The branch bark texture was classed according to the degree of roughness, fissuring and flakiness. Each category was independently scored by two observers as being absent, weakly developed or strongly developed (score 1–3). The aspect and inclination of each branch was measured using a standard compass with clinometer. Aspect was measured by following the general orientation of each branch. Branches that had unusual growth forms (e.g. zigzag growth) were divided into multiple sections and each section recorded individually for aspect. Total bryophyte cover and total lichen cover (all life-forms) were estimated for each branch and bole, using a 0–100 % scale with 5 % intervals.

The position of each dependent plant individual was recorded by dividing the branch into adaxial side (i.e. upper half-cylinder) and abaxial side. The positions of hemi-epiphytes, climbers, nomadic vines and mistletoes were measured by identifying as far as possible the position of the initial rooting point. Where it was not possible to determine a germination point, we estimated the total area they occupied and recorded the aspects they covered on the tree.

### Hurricane model

As described in Batke *et al*. (2014) and summarized in Batke and Kelly (2014), we used past hurricane data to model the impact of hurricanes at CNP. In the work presented here, we compared the different model solutions (expressed as exposure vulnerability site score—EVSS) to diversity and composition data for canopy-dependent plants in CNP at a tree level.

### Data analysis

The community analysis at plot and tree level was performed on the whole-community data set and was not divided by life-forms (the low number of occurrences in some life-form groups would have yielded unreliable ordinations scores). On a branch level, however, we analysed the community data separately by life-forms and by taxonomic families. Juvenile individuals that could not be identified sufficiently and accidental-dependent plants were excluded from the community analysis. Individuals that could not be determined to species rank but that clearly represented single taxonomic entities were treated as separate species. To avoid multicollinearity, only environmental variables were selected with an *R*^2^ < 0.75 (Heikkinen *et al*. 2006). When two variables were strongly correlated (i.e. *R*^2^ > 0.75) the variable with the higher variance inflation factor was retained (O'Brien 2007). Circular data (i.e. aspect) were transformed using trigonometric functions prior to analysis (Austin 2007). Branch inclination was divided into three classes after Ingram and Nadkarni (1993): angle class one, 0–30°, angle class two, 31–60° and angle class three, 61–90°.

A distance-based redundancy analysis (db-RDA) was used to detect linear relationships between dissimilarity matrices between plots, trees and branches. The Euclidean distance matrix was calculated in ArcGIS 10 and transformed using Sturges' rule as described by Wolf *et al*. (2009) and axis scores were calculated using principle coordinates analysis. The db-RDA was run with a Euclidean distance measure and 999 permutations for each matrix combination (i.e. the environmental, the geographical distance and the combined environmental and geographical distance matrix). The relationship between axis scores was determined using randomization tests at a *P*-value of 0.05. This ordination method allowed identifying how much variation in the species community matrix was explained by different environmental variables and geographic distance.

The analyses were performed in the statistical program ‘R’ version 2.15.0 (R Developing Core Team 2011), Data Desk version 6.1 (Velleman and John 1996) and PC-ORD version 6.0 (McCune and Mefford 2011).

## Results

**Figure 2.**
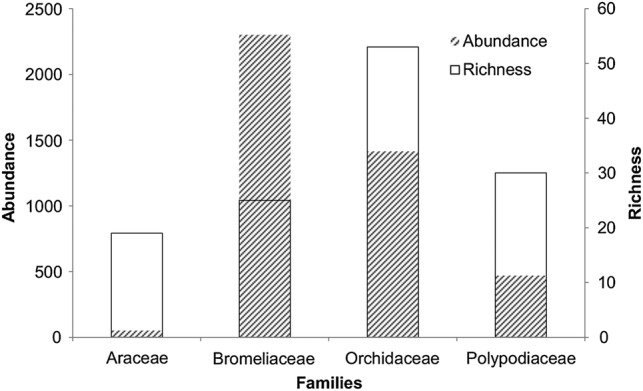
The four mechanically dependent families with the highest species richness and abundance of individuals in Cusuco National Park.

A total of 7074 individuals of mechanically dependent species from 60 host trees were identified. The majority of dependent plants censused (71.5 %) were infertile (having neither flowers nor fruit). Individuals (98.9 %) were identified to family, 95.1 % to genus and 69.8 % to species level. An additional 10.9 % of individuals apparently matched a particular species but were not confirmed as the same (and are distinguished as ‘cf.’). From the 7074 individuals, a total of 214 species from 90 different genera and 43 different families were identified [see Batke *et al*. (2015) for a full account of the species]. The four families with the highest species richness and abundance were Orchidaceae, Polypodiaceae, Bromeliaceae and Araceae (Fig. 2). The life-form group with the highest species richness and abundance was holo-epiphytes, followed by climbers, nomadic vines, primary hemi-epiphytes, mistletoes and accidental epiphytes (Table 1). Although stranglers were observed in CNP [*Ficus* (Moraceae), three species], none were found on the sampled trees.

**Table 1.** Overall life-form composition of the dependent flora for CNP. The total species richness and abundance of individuals are given for all high (1800–2000 m a.s.l.; *n* = 5) and low (1300–1450 m a.s.l.; *n* = 5) elevation plots and the whole study area (*n* = 10).

Life-form	Abundance		Richness	
	Low elevation	High elevation	Low elevation	High elevation
Holo-epiphytes	2613	3910	82	85
Hemi-epiphytes	37	310	4	18
Nomadic vines	28	24	11	13
Mistletoes	36	18	2	4
Climbers	41	45	22	9
Accidental epiphytes	7	5	3	1
Stranglers	0	0	0	0
Sub-total	2762	4312	124	130
Total	7074		214

### Community analysis

The db-RDA revealed strong linear dependency between matrices at a plot, tree and branch level. At a plot level 36.9 % of variance was explained by environmental variables and 41.3 % by geographical distance between plots, with a shared variance of 49.1 % (Table 2). The two most important environmental factors that were driving the community dissimilarity were mean vapour pressure deficit (VPD) and elevation **[see Supporting Information—Table S1]**.

**Table 2.** The db-RDA variance explained by environmental variables and geographical distance of the mechanically dependent plant community in CNP. nd, data insufficient for analysis. The degree of overall relationships between predictor and response matrices was determined at *P < 0.05, **P < 0.01 and ***P < 0.001. Low elevation = 1300–1450 m a.s.l.; high elevation = 1800–2000 m a.s.l.

Level	Environmental variance (%)	Geographic variance (%)	Shared variance (%)
Plot	36.9*	41.3	49.1*
Tree	7.8*	8.3***	11***
Low elevation	24.3**	26***	30.4**
High elevation	14	17.1***	18.2**
Branch	1.5***	nd	nd

At a tree level, 11 % of variance was shared by geographical distance between trees (8.3 %) and environmental variables (7.8 %; Table 2). As elevation had a strong overall effect on the vegetation dissimilarity in the ordination space at a plot level **[see Supporting Information—Table S1]**, the species data were analysed separately at a tree level for low (1300–1450 m a.s.l.) and high (1800–2000 m a.s.l.) elevation sites. This was done to identify other environmental variables that might affect the vegetation composition between trees in the absence of large elevation ranges. Following the separation of individual trees into low- and high-elevation sites, the contributions of environmental variables increased (low elevation: 24.3 %; high elevation: 14 %; Table 2). At low-elevation sites, the two most important environmental variables were canopy openness and past hurricane damage from south-easterly winds **[see Supporting Information—Table S2]**. At high-elevation sites, the two most important environmental variables were canopy openness and past hurricane damage from southerly winds **[see Supporting Information—Table S2]**.

At a branch level, only 1.5 % of variance was explained by environmental variables (Table 2). The two most important environmental factors that were driving the community, family and life-form dissimilarity were branch surface area and the cover of epiphytic bryophytes **[see Supporting Information—Table S3]**. Bryophyte cover was significantly lower on branches located in trees that were more impacted by hurricanes (EVSS 1 = 51.7 %; EVSS 5 = 20.9 %; DF = 421, *R*^2^ = 0.68, *P* < 0.01).

### Between-tree variation

**Figure 3.**
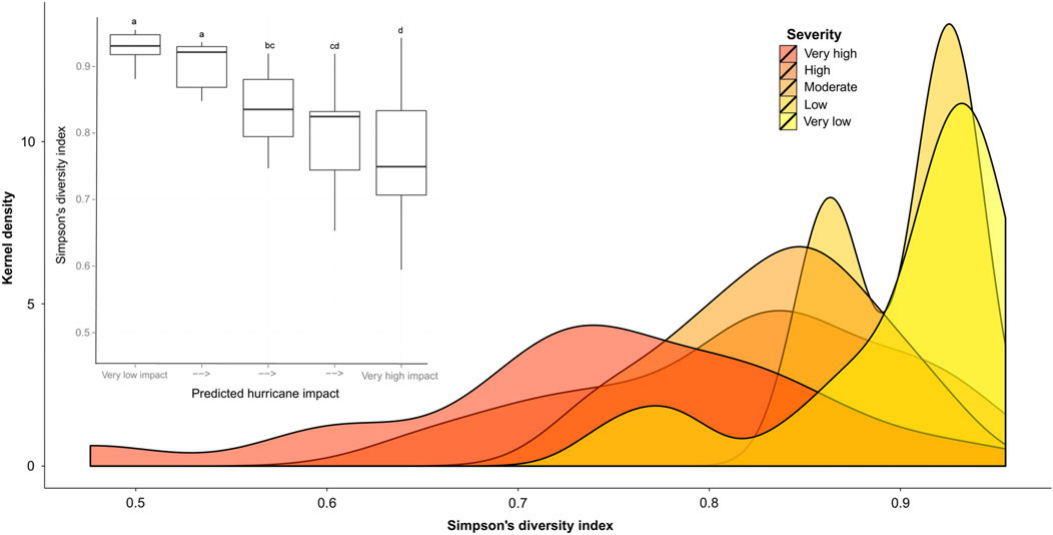
Boxplot and kernel density plot of the Simpson's diversity index in relation to predicted hurricane impact for the EVSS south solution (southerly wind direction = best fit solution). Significant differences (*α* = 0.05) between impact levels are indicated by letters.

Dependent plant diversity decreased with increasing predicted hurricane damage (Simpson's diversity index, *ρ* = −0.68, *P* < 0.01 and species richness, *ρ* = −0.57, *P* < 0.01; Fig. 3). Similarly, species richness and diversity were negatively correlated with increased canopy openness (richness: *ρ* = −0.369, DF = 59, *P* < 0.01; Simpson's diversity: *ρ* = −0.531, DF = 59, *P* < 0.01). Pairwise comparisons revealed that closed canopies were significantly different (both in terms of diversity and species richness) compared with intermediate and open canopies. However, intermediate canopies did not differ significantly from open canopies. Furthermore, dependent plant diversity (but not richness) was correlated with tree type: conifer trees had a significantly lower dependent plant diversity compared with angiosperm trees (Simpson's diversity: conifer *D*′ = 0.79, *ρ* = −0.311, DF = 17, *R*^2^ = 0.84, *P* = 0.03, versus angiosperms *D*′ = 0.84, richness: *ρ* = −0.221, DF = 41, *R*^2^ = 0.01, *P* > 0.05).

Species richness of holo-epiphytes (*t* = −6.78, DF = 97, *R*^2^ = 0.19, *P* < 0.01) and primary hemi-epiphytes (*t* = −2.89, DF = 17, *R*^2^ = 0.42, *P* < 0.01) were negatively related with increasing hurricane impact from southerly winds. All other comparisons of richness with life-forms were non-significant (climbers: *t* = 0.13, DF = 16, *P* > 0.05; nomadic vines: *t* = −1.1, DF = 11, *P* > 0.05; mistletoes: *t* = −0.25, DF = 3, *P* > 0.05). Abundance of different life-forms did not change with hurricane impact (holo-epiphytes: *t* = −1.2, DF = 6522, *P* > 0.05; climbers: *t* = 0.03, DF = 85, *P* > 0.05; nomadic vines: *t* = −0.11, DF = 51, *P* > 0.05; mistletoes: *t* = −0.05, DF = 53, *P* > 0.05; primary hemi-epiphytes: *t* = −0.86, DF = 346, *P* > 0.05).

### Within-tree variation

**Figure 4.**
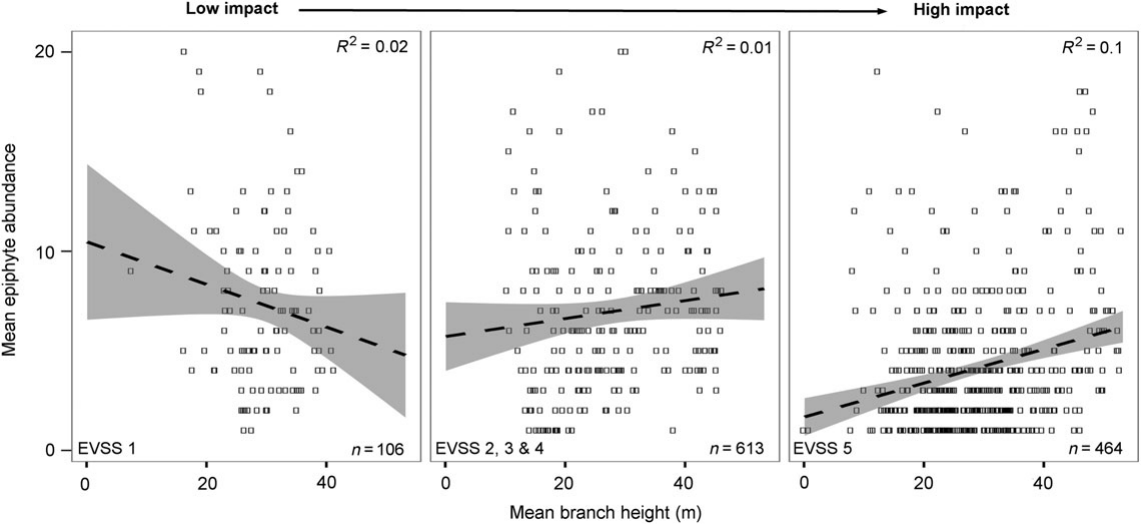
Mean dependent plant abundance correlated to mean branch height across the different hurricane impact levels. The dashed line represents the line of best fit and the grey-shaded areas are the 95 % confidence limits. The number of branches that were included in the analysis between the different impact levels is noted at the bottom of each graph. Note that only a total of 60 branches were randomly selected for the final analysis.

The abundance of dependent plants with distance to the tree centre did not change with increased hurricane impact (*P* > 0.05). However, the relationship between dependent plant abundance and height in the tree varied in relation to hurricane impact level (*t* = 2.02, DF = 1182, *R*^2^ = 0.1, *P* < 0.05). Dependent plant abundance decreased with level of hurricane impact on a branch level. Overall, abundance was highest on branches located on the lower parts of the tree in areas where hurricane impact was low, and lowest on branches located on the lower parts of the tree in areas where hurricane impact was high. However, the data fit was low (Fig. 4).

Following closer investigation, it became apparent that the observed patterns in Fig. 4 could be mainly attributed to abundance shifts of individual species. For example, abundance of *Tillandsia vicentina* (a bromeliad) increased with increasing hurricane impact and the abundance shifted from the lower and middle canopy (20–40 m) to middle and upper canopy (40–60 m). Furthermore, the drought resistance bromeliad *Tillandsia seleriana* was absent at very low and low impact levels. The highest abundance of this species was observed at very high impact levels, at the middle canopy (20–30 m). The abundance of the fern *Pleopeltis mexicana* was highest at branches located between 20 and 40 m in low impact levels; it was almost completely absent at medium, high and very high impact levels. The tank bromeliads *Catopsis hahnii* and *C. floribunda* were completely absent at very low impact levels but increased towards high impact levels; however, *C. hahnii* was absent at very high impact levels. Most individuals of *C. hahnii* were found between 40 and 60 m at medium impact levels and between 40 and 50 m at high impact levels. At very low impact levels, the atmospheric bromeliad *Tillandsia butzii* was almost completely absent throughout the canopy. Abundance increased substantially with hurricane impact. At low impact levels *T. butzii* abundance was highest at the middle and upper canopy (40–50 m); at high and very high impact levels, the highest abundance shifted towards lower canopy branches (20–40 m). Finally, the abundance of the tank bromeliad *Werauhia werckleana* was highest, across the different impact levels, in the lower canopy (0–20 m).

### Within-branch variation

Dependent plant abundance on the adaxial branch surface decreased with increasing hurricane incidence, however this was not significant at a community level (*G* = 22.97, DF = 2396, *P* > 0.05). Moreover, there was no difference in abundance on the abaxial branch surface with increasing hurricane impact at a community level (*G* = 41.1, DF = 2396, *P* > 0.05). At a family level, abundance did change with hurricane impact (*P* < 0.01). For example, Bromeliaceae abundance at low impact sites was highest both on adaxial branch surfaces (72.8 %) and on abaxial branch surfaces (50.8 %). Orchidaceae abundance showed a reversed pattern. On high impacted sites, orchid abundance was highest on the adaxial branch surfaces (89.9 %) and lowest on the abaxial surfaces (10.2 %).

## Discussion

Studies that investigate hurricane effects on dependent plants have mostly been descriptive in scope (Loope *et al*. 1994) and are commonly limited to a particular group of dependent plants (e.g. holo-epiphytes), limited to a particular section within the canopy (Goode and Allen 2008) and/or restricted to only one disturbance event (Rodriguez-Robles *et al*. 1990; Mújica *et al*. 2013). Here we investigate how the community composition and diversity of a dependent flora of 214 species, sampled along the whole vertical forest profile, changed in relation to past hurricane impacts, to different environmental and structural variables, and to geographical distance between host trees.

We identified different variables at plot, tree and branch levels that influenced the community dissimilarity of mechanically dependent plants across our the study site. The most important variables at a landscape level were elevation and mean VPD; at an individual tree level, canopy openness and hurricane exposure and at a branch level, branch surface area and bryophyte cover. The shared contribution of environmental and structural variables in determining the composition of dependent plants decreased from plot level (49.1 %), to tree level (∼18–30 %). Our results are in line with the view that dependent plant community composition on a landscape level is mainly driven by differences in plot elevation (Frahm and Gradstein 1991; Wolf 1993; Hietz and Hietz-Seifert 1995; Kessler 2000), climate (Benzing 1998*a*), aspect and degree of exposure. On a local level, on the other hand, characteristics such as host properties and geographical distance between the host trees have been suggested to be more important (Wolf *et al*. 2009).

### Plot-level variation

The strongest vegetation clustering was observed between low- (1300–1450 m a.s.l.) and high-elevation (1800–2000 m a.s.l.) sites. In previous work we showed that 15 % of the annual variance in temperature and relative air humidity (expressed as VPD) in CNP was explained by differences in altitude (Batke and Kelly 2014). At higher elevation the occurrence of clouds and rain is more frequent and the air has a lower water-holding capacity because of the decrease in temperature. As many mechanically dependent plants depend on the availability of water (Benzing 1987, 1998*b*; Zotz *et al*. 2010), the strong difference in community composition between low- and high-elevation sites is likely to reflect physiological limitations and habitat preferences of particular dependent species. Species that are more tolerant to lower levels of humidity and higher levels of temperature are likely to occur more frequently at low-elevation sites (Gentry and Dodson 1987; Wolf and Flamenco-S 2003).

### Tree-level variation

At tree level, across the low- and high-elevation sites, mechanically dependent plants responded strongly to different levels of hurricane impact, canopy openness and host tree life-form (broadleaved angiosperm versus needle-leaved conifer). We detected significant diversity and compositional effects on mechanically dependent plant species along an impact gradient of past hurricanes. Across the elevation range, dependent plant diversity and species richness were negatively correlated with increased predicted past hurricane impact and canopy openness. Open canopies can increase stress to the dependent plants through maximizing wind, sun and drought exposure and thus reducing favourable conditions for growth and survival (Zotz and Hietz 2001). More open canopies following wind damage can result in direct community responses (e.g. increased mortality) or indirect community responses [e.g. changes affecting germination and establishment rates (Graham and Andrade 2004; Mondragon and Maria Calvo-Irabien 2006)]. This was demonstrated by Kartzinel *et al*. (2013), who found that canopy openness was strongly negatively associated with germination rates in neotropical orchids. This was because disturbed sites did not provide favourable microsites for the epiphyte and its associated mycorrhizal community.

The slow recovery rates of dependent plant communities reflect high juvenile mortality (Werner and Gradstein 2008), slow growth rate (Laube and Zotz 2003) and slowness to reach maturity (Zotz 1995; Schmidt and Zotz 2002). These factors, taken together with the recovery response rate of the forest canopy in relation to a relatively low frequency of recurring hurricane events (Batke *et al*. 2014), have combined to produce a patchy disturbance mosaic within the forest in CNP. A similar picture was obtained for swamp cypress (*Taxodium distichum*) forest in Florida (Oberbauer *et al*. 1996). Loope *et al*. (1994) reported that vascular epiphytes suffered the highest mortality rates of all plant groups (∼90 % mortality) in upland and swamp forest in Florida following hurricane Andrew in 1992. They also noted that the epiphyte response to the hurricane damage varied among epiphytic groups, which resulted in an asymmetrical community response. For example, many *Tillandsia* species suffered significant sun damage compared with other groups such as orchids, which seemed less sensitive to increased solar radiation. Likewise, Goode and Allen (2008) found that epiphyte abundance was significantly reduced in a dry forest in Mexico following hurricane Wilma in 2005; the epiphyte species composition remained, however, similar to pre-hurricane conditions.

Across the range of dependent life-forms included in our study, we recorded significant effects of increased hurricane impact only for holo- and hemi-epiphyte richness. This is, at least in part, a reflection of our research design; sampling was less adequate for species with large individuals, of which only very small numbers can be expected on any one tree—as was the case for most climbers. Closer investigation revealed that the decrease in richness was associated with particular taxonomic groups within life-form categories, rather than with particular life-forms *per se*. For example, species of the holo-epiphyte-dominated family Orchidaceae showed positive responses to increased hurricane impact, whereas many fern species showed negative responses. As discussed by Loope *et al*. (1994), the increase in orchid richness is most likely the result of higher light radiation, probably as a result of increased canopy openness. Although orchids are very susceptible to hurricane damage (Rodriguez-Robles *et al*. 1990), particularly after the direct passing of hurricane winds (Migenis and Ackerman 1993), they are often superior to other epiphytes in tolerating long desiccation periods [they have the ability to store water in most parts of the plant body (Ng and Hew 2000)].

### Branch-level variation

Dependent plant abundance shifted along a gradient of hurricane impact. The shift in abundance with increasing hurricane impact was mainly a reflection of abundance changes among individual species but was compounded by shifts in community composition. For example, the abundance of many orchid species increased with hurricane impact on the upper canopy. The differences in response among species were possibly a reflection of the different niche requirements and differing degrees of susceptibility to disturbance (e.g. to changes in the microclimate) among species. The tank bromeliads *C. hahnii* and *C. floribunda*, for example, occurred frequently in the upper canopy in high impact sites. These species have been reported to be relatively well adapted to exposed conditions [presence of tank, medium density of trichomes (absorptive scales) and short time-span to maturity (Benzing and Renfrow 1971; Hietz *et al*. 2002)]. Dependent plant species that are less well adapted to exposed conditions are thus expected to diminish or shift in their distribution to more favourable microsites. In the case of the mesic *W. werckleana*, we found that the species occurred at heights between 0 and 20 m across all levels of hurricane impact; but with a lower abundance on sites that were more impacted by hurricanes. As *W. werckleana* is limited to the lower canopy, possibly as a result of physiological limitation (Pittendrigh 1948; Reyes-García *et al*. 2008), any further negative shift in the canopy microclimate (e.g. as a result of more severe canopy damage) could result in the loss of this species from such sites. Losses would be concentrated among individuals that are small in size, as they are most susceptible to changes in the micro-environment (Zotz and Thomas 1999).

The adaxial surface of a branch is more vulnerable to changes in microclimatic conditions than the abaxial surface. Thus, we would expect epiphytes to be less abundant on the adaxial surfaces in areas that have been impacted more as a result of wind damage. These branches are also more likely to be stripped of epiphytes due to their exposed position. We found that the abundance of several holo-epiphyte families was significantly different among branches exposed to different levels of predicted hurricane impact. Branches that have been stripped of dependent plants during severe gusts are likely to be less favourable for some species during early colonization, due to altered habitat conditions (e.g. the absence of organic substrata such as bryophytes). Post-perturbation recovery and colonization would most likely be the main community composition drivers on these branches (Turner *et al*. 1998). Nadkarni (2000) demonstrated that, after experimental branch stripping of all organic material, epiphyte colonization took place upwards from the abaxial branch surface. She suggested that the higher abundance of bryophytes and the resulting higher water-retention capacity of the abaxial branch surfaces, due to shading effects of the branch itself, had made these sites more suitable for colonization. During ontogeny, epiphytes are expected to grow towards the adaxial surface of the branch, as plants are increasingly relieved from water stress due to their larger size (Zotz and Vollrath 2002). Although we did not assess colonization, we found that mean bryophyte cover was significantly reduced on high impact sites (by ∼30 %).

## Conclusion

In conclusion, dependent plant communities in CNP have been affected by the impact of past hurricanes. Direct effects were not observed, as the time-lapse between the fieldwork (2012–13) and the last major hurricane impact (1998 [Batke *et al*. 2014]) was too great. Species diversity and floristic composition both showed indirect effects derived from hurricane damage. The observed community shift, and the lower observed diversity of dependent plants with increasing hurricane impact, can most likely be attributed to structural and micro-environmental alteration of the forest canopy [e.g. branch breakage (Batke and Kelly 2014)]. Several studies that have investigated structural and micro-environmental canopy alterations (e.g. due to logging) have demonstrated that dependent plant communities will change in response to these modifications. For example, Werner and Gradstein (2009) found that epiphytes were constrained by changes in the canopy microclimate along a human disturbance gradient and that these changes were the result of structural alterations to the forest (e.g. increased canopy openness and increased edge effects). The dependent plant response we observed at CNP is much more noticeable at an individual species level than on a life-form level; this reflects the fact that ecophysiological responses can be highly variable even between ecologically closely linked taxa (Saldaña *et al*. 2005).

Canopies that were more affected by hurricanes had a more open canopy, higher VPD (Batke and Kelly 2014) and a lower diversity of dependent plants. The change in the canopy micro-environment with increasing hurricane impact thus had negative effects on both non-vascular epiphyte cover and vascular-dependent plant abundance at a branch level. As forest canopies are living, self-maintaining structures that may quickly recover from perturbation events, it can be predicted that micro-environmental conditions may also recuperate relatively swiftly. Certain mechanically dependent species will gain competitive advantage, eventually restoring dependent plant community structure across the canopy to a situation reflecting pre-hurricane impact patterns (Goode and Allen 2008). It is likely that the pre-hurricane dynamic equilibrium may be rather rapidly regained, as has been demonstrated in a number of other taxa (Waide 1991*a*, *b*; Wunderle *et al*. 1992).

Direct effects on mechanically dependent plants (e.g. wind-throw and tissue damage) can mostly be measured only directly after the passing of hurricane winds, whereas indirect effects due to structural and microclimatic alterations of the forest canopy are likely to be measureable for much longer. In regions where hurricanes are more frequent, effects on the dependent plant community will be much stronger and possibly result in long-term decline in diversity as shown by Mújica *et al*. (2013) and Raventós *et al*. (2015). For example, Raventós *et al*. (2015) reported that the population viability of the orchid *Dendrophylax lindenii* in Cuba (an area that is frequently affected by hurricanes) was negative (*λ* = 0.975). Their population viability simulations suggested that hurricanes could result in the near extinction of this orchid (∼25 years), if the local occurrence of disturbances are high (>14 %). Management and conservation efforts in relation to post-hurricane-dependent plant communities therefore need to focus on the identification of suitable host trees that have not been damaged by the hurricane winds. As in the case of CNP, forest conservation should not only consider the protection of primary forest sites but also incorporate sites that are less likely to be affected by hurricane storms. These sites can act as refuges for dependent plants and increase the rates of recolonization following hurricane damage (Löbel *et al*. 2006). This could be particularly important in CNP, where many dependent plants appear to be very scare and/or patchy in their distribution.

## Sources of Funding

We thank Trinity College Dublin, The Rufford Foundation (#13405-1) and Operation Wallacea for their financial support.

## Contributions by the Authors

This project was conceived and designed by both authors. The data collection and analysis was performed by the first author. The second author aided during the species identification. The article was written and edited by both authors.

## Conflict of Interest Statement

None declared.

## Supporting Information

The following additional information is available in the online version of this article –

**Table S1.** Plot dependent-plant NMS ordination axis scores correlated to environmental variables using Spearman's rank correlation coefficient.

**Table S2.** Tree dependent-plant NMS ordination axis scores correlated to environmental variables using Spearman's rank correlation coefficient.

**Table S3.** Branch dependent-plant NMS ordination axis scores correlated to environmental variables using Spearman's rank correlation coefficient.

Additional Information
