# Supplementary material for: Changes in the distribution of mechanically dependent plants along a gradient of past hurricane impact
Source: AoB Plants. 2015 Aug 18;7:plv096. doi: 10.1093/aobpla/plv096 (PMC4584959; doi:10.1093/aobpla/plv096)
Supplement: Additional Information [file supp_7_plv096_index.html]

Changes in the distribution of mechanically dependent plants along a gradient of past hurricane impact — Additional Information 

# Changes in the distribution of mechanically dependent plants along a gradient of past hurricane impact

## Additional Information

Additional Information

- Supporting Information - Table 1 - docx file
- Supporting Information - Table 2 - docx file
- Supporting Information - Table 3 - docx file
