# Supplementary material for: Changes in the distribution of mechanically dependent plants along a gradient of past hurricane impact
Source: AoB Plants. 2015 Aug 18;7:plv096. doi: 10.1093/aobpla/plv096 (PMC4584959; doi:10.1093/aobpla/plv096)
Supplement: Additional Information [file supp_plv096_plv096supp_table1.docx]

**Table 1** Plot dependent-plant db-RDA ordination axis scores correlated to environmental variables using linear regression. Individual plots were used as a sampling unit and an ordinations was run at a community levels. A Euclidean distance measure was used to identify the percent of variance in the distance matrix for each axis. Only coefficient values that were significant are shown (P < 0.05). ns = non-significant.

| **Level** | **Variables** | **Community** | | |
| --- | --- | --- | --- | --- |
| **Plot** (n = 10) |  |  |  |  |
|  |  | Axis1 | Axis2 | Axis3 |
|  | Mean VPD | 0.221 | -0.006 | 0.905 |
|  | Elevation | -0.534 | 0.117 | -0.767 |
|  | Aspect | ns | ns | ns |
|  | Slope | ns | ns | ns |
|  | Exposure | ns | ns | ns |
