# Supplementary material for: Changes in the distribution of mechanically dependent plants along a gradient of past hurricane impact
Source: AoB Plants. 2015 Aug 18;7:plv096. doi: 10.1093/aobpla/plv096 (PMC4584959; doi:10.1093/aobpla/plv096)
Supplement: Additional Information [file supp_plv096_plv096supp_table2.docx]

**Table 2** Tree dependent-plant db-RDA ordination axis scores correlated to environmental variables using linear regression. Individual trees were used as a sampling unit and an ordinations was run at a community levels. Low and high elevation trees were analyzed separately. A Euclidean distance measure was used to identify the percent of variance in the distance matrix for each axis. Only coefficient values that were significant are shown (P < 0.05). ns = non-significant.

| **Level** | **Variables** | **Community** | | |
| --- | --- | --- | --- | --- |
| **Tree** (n = 60) |  |  |  |  |
|  |  | Axis1 | Axis2 | Axis3 |
| Low elevation (n = 30) |  |  |  |  |
|  | Canopy openness | ns | -0.80 | ns |
|  | EVSS (SE) | -0.77 | ns | ns |
|  | Tree surface area | ns | -0.33 | 0.75 |
|  | Canopy extent (N) | -0.25 | 0.11 | 0.64 |
|  | Tree height | -0.64 | -0.15 | 0.30 |
|  | Canopy extent (E) | ns | -0.28 | 0.55 |
|  | Tree circumference | -0.31 | -0.22 | 0.59 |
|  | Tree type | -0.53 | -0.35 | ns |
|  | Canopy extent (W) | -0.36 | 0.47 | 0.29 |
|  | Canopy extent (S) | ns | ns | ns |
|  | EVSS (S) | ns | ns | ns |
| High elevation (n = 30) |  |  |  |  |
|  | Canopy openness | -0.52 | 0.70 | ns |
|  | EVSS (S) | ns | 0.58 | ns |
|  | Tree height | 0.27 | -0.50 | -0.51 |
|  | EVSS (SE) | ns | 0.46 | ns |
|  | Tree surface area | 0.15 | -0.31 | -0.48 |
|  | Tree circumference | 0.26 | ns | -0.48 |
|  | Canopy extent (W) | 0.15 | ns | -0.47 |
|  | Tree type | ns | 0.40 | ns |
|  | Canopy extent (S) | 0.33 | ns | -0.36 |
|  | Canopy extent (N) | 0.33 | 0.20 | -0.13 |
|  | Canopy extent (E) | 0.12 | 0.20 | -0.22 |
