# Supplementary material for: Changes in the distribution of mechanically dependent plants along a gradient of past hurricane impact
Source: AoB Plants. 2015 Aug 18;7:plv096. doi: 10.1093/aobpla/plv096 (PMC4584959; doi:10.1093/aobpla/plv096)
Supplement: Additional Information [file supp_plv096_plv096supp_table3.docx]

**Table 3** Branch dependent-plant db-RDA ordination axis scores correlated to environmental variables using linear regression. Individual branches were used as a sampling unit and an ordinations was run at a community, family and life-form levels. A Euclidean distance measure was used to identify the percent of variance in the distance matrix for each axis. Only coefficient values that were significant are shown (P < 0.05). ns = non-significant.

| **Level** |  | **Community** | | | **Family** | | | **Life-form** | | |
| --- | --- | --- | --- | --- | --- | --- | --- | --- | --- | --- |
| **Branch** (n = 1108) |  |  |  |  |  |  |  |  |  |  |
|  |  | Axis1 | Axis2 | Axis3 | Axis1 | Axis2 | Axis3 | Axis1 | Axis2 | Axis3 |
|  | Branch surface area | -0.45 | -0.29 | ns | 0.15 | 0.42 | -0.27 | -0.46 | ns | ns |
|  | Bryophyte cover | -0.46 | 0.39 | ns | 0.23 | 0.44 | -0.24 | 0.19 | 0.20 | ns |
|  | Lichen cover | 0.36 | 0.18 | ns | ns | -0.32 | ns | 0.31 | ns | ns |
|  | Bark roughness | -0.19 | ns | ns | 0.29 | 0.15 | ns | -0.19 | ns | ns |
|  | Bark flakiness | ns | ns | ns | -0.21 | ns | ns | ns | 0.16 | ns |
|  | Branch inclination | ns | 0.16 | -0.42 | ns | ns | 0.21 | 0.10 | ns | ns |
|  | Bark fissuring | ns | ns | ns | ns | ns | -0.16 | 0.12 | ns | ns |
|  | Branch aspect | ns | ns | ns | ns | ns | ns | ns | ns | ns |
